# New model of college physical education teaching based on the algorithm and data structure of flipped classroom and OBE

**DOI:** 10.1016/j.heliyon.2024.e31368

**Published:** 2024-05-18

**Authors:** Yanjun Kong, Weihong Wang, Bakhrom Rajabov

**Affiliations:** a School of Physical Education, Hebei Normal University for Nationalities, Chengde, 067000, Hebei, China; b School of Physical Education, Sichuan University, Chengdu, 610065, Sichuan, China; c School of Transportation and Logistics, Southwest Jiaotong University of China, Chengdu, 610031, Sichuan, China

**Keywords:** Wireless network, Flipped classroom, Outcome-based education, Algorithm and data structure, College physical education

## Abstract

Although college physical education (PE) is a compulsory course in college teaching, due to the openness of assessment standards and students studying to pass the final exam, college PE has been undervalued, so this paper aims to explore the new model of college PE teaching. In response, this paper took the air volleyball course as an example and redesigned the teaching of college PE based on the theory of flipped classroom and outcomes-based education (OBE). This paper also proposed a personalised learning system for college sports based on genetic algorithm (GA) and data structure, greatly improving college PE's autonomous learning ability and willingness. In the design of the teaching model, this paper compared and analysed the teaching model combining flipped classroom and OBE with flipped classroom teaching model, OBE teaching model and traditional teaching model. A one-semester investigation was conducted by selecting 40 students who took the air volleyball PE course at Hebei Normal University for Nationalities. The 40 students were divided into four groups, and their learning after one semester was compared. The experimental results showed that, compared with the traditional teaching model group, the outcome-based education and flipped classroom education group's performance of hitting the ball, passing the ball, spiking the ball, and serving the ball increased by 3.8 %, 14.3 %, 20.8 %, and 10.3 %, respectively. This suggested that the new college physical education teaching model based on flipped classrooms and OBE has a good teaching effect and can be used as a reference and help for others.

## 1 Introduction

Unlike wired networks, wireless networks break through the limitations of wired networks. They can connect people at any time through wireless signals. Their network expansion performance is relatively strong, and they can effectively achieve network work expansion and configuration settings. Users will also become more efficient and convenient when accessing information. The wireless network not only expands the space range of people who use the network but also improves the efficiency of the network. The traditional PE teaching model focuses on implementing teaching methods and means and only pays attention to the completion rate and pass rate of students' learning tasks [1]. In teaching college PE, such a teaching model can allow students to understand the basic theoretical knowledge of air volleyball and master basic sports skills. However, for the employment of students majoring in PE, this is far from meeting modern society's needs for air volleyball professionals. Especially in the process of air volleyball gradually getting on the right track, the demand for air volleyball professionals in society and school sports increases greatly, and the requirements continue to increase. Cultivating talent in PE majors is a powerful guarantee for supplying talent for developing school sports and social sports in China. The general public's physical and mental health and physical fitness need guarantee and support, and China's transformation from a sports power to a sports power requires high-quality reserve talents. Therefore, this paper must study the new model of college PE based on flipped classrooms and OBE. The personalised learning model of genetic algorithms has introduced new ideas into physical education. By optimising the teaching mode, students' learning needs can be met individually, which provides innovative exploration for improving the teaching effect and cultivating students' comprehensive quality. This paper has important theoretical and practical value for promoting the innovation of physical education teaching mode and improving students' learning experience and overall quality.

College PE is a compulsory course in college education. However, due to the lack of unified evaluation standards and teaching models, students have not valued it for a long time. In this regard, many scholars have studied the new model of college PE. Wang et al. [2] through the reconstruction of the reform of teaching methods, we should integrate the relationship between modern technology and sports, build a bridge between information technology and traditional teaching with the help of the classroom platform, realise the transformation of sports technology and skills from imparting to learning, realise the lifelong sharing of resources with the help of the public platform, create a precedent for lifelong sports exercise classes, and fully realise the function of university sports to serve the society. Xin and Wang [3] investigated the current situation of PE in colleges and universities under the cloud computing environment, analysed and summarised the problems existing in the process of PE in colleges and universities, and gave corresponding countermeasures and suggestions. Starting from the significance and characteristics of the experiential teaching model, Wang [4] expounded the implementation significance and existing problems of the current PE teaching model and finally explored effective application strategies. To meet the market demand for social sports, Yu et al. [5] optimised and adjusted it based on the original "dual system" teaching model for sports talents in colleges and universities, combined with the advanced talent introduction model, to establish a new college sports talent training system and optimise the existing talent training model. To better implement the student-oriented concept and promote the growth and development of college students, Wang and Tang [6] studied the path and method of building a team of college students' growth mentors based on political work. Their research not only started with technology and introduced information technology but also started with teaching methods and put forward many new models and suggestions. However, their research lacked thinking about student groups and did not consider students as the main object.

The flipped classroom and OBE theory are more commonly used methods in modern teaching, and many scholars have conducted research on them. Song [7] discussed the problems in logistics teaching and the urgency of curriculum reform. He introduced the concept of outcomes-based education (OBE) into the logistics teaching process, reversed the design of teaching based on results, and implemented the teaching design as a flipped classroom. Kadam and Sawant [8] studied the teaching mode of communication skills for senior students, and they used the OBE concept and flipped classroom to optimise the teaching model. The effectiveness of flipped classrooms is currently debated due to conflicting results from different studies. Sajid et al. [9] aimed to evaluate the efficacy and acceptability of the flipped classroom in undergraduate medical education at the Faculty of Medicine, Alpha Sal University. Tavares et al. [10] conducted a systematic literature review on the flipped classroom methodology with a focus on K-12 education. They proposed model recommendations for using digital information and communication technologies as a methodology support tool for the flipped classroom. Liu et al. [11] analysed the problems and challenges in the epidemic environment from the perspectives of teachers, students and technology and proposed the teaching model of "re-flipped classroom" and the teaching mode of "SPOC + MOOC + live broadcast". Combining OBE with mind mapping, the teaching evaluation was introduced into teaching design, and finally, implementation suggestions were put forward to ensure the quality of teaching. However, there were few studies on applying flipped classrooms and OBE concepts to PE, and the research was not deep enough.

The innovations of this paper are as follows. For college PE, this paper starts with the well-known air volleyball and selects experimental objects from various majors in the school, thus ensuring the universality of the experimental results in this paper. In the experiment, this paper not only compares and analyses the new teaching model designed in this paper with the traditional teaching model but also compares it with the new teaching model of flipped classroom theory and OBE concept.

## 2 New model of college PE teaching

### 2.1 Concept of flipped classroom and OBE

**Fig. 1:**
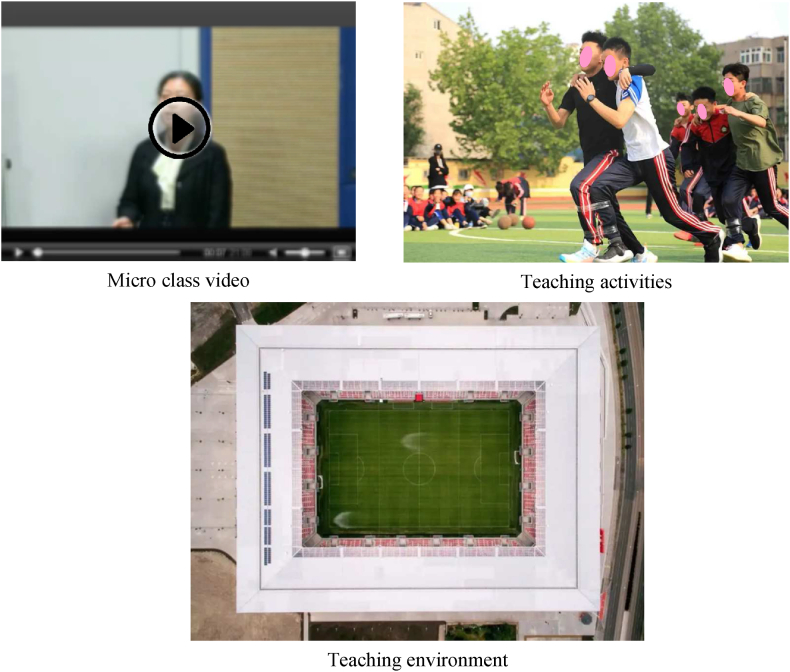
Three elements of a flipped classroom.

The flipped classroom emphasises that students are the main body and need the support of micro-lectures, a teaching environment, and teaching activities, as shown in Fig. 1.

**Fig. 2:**
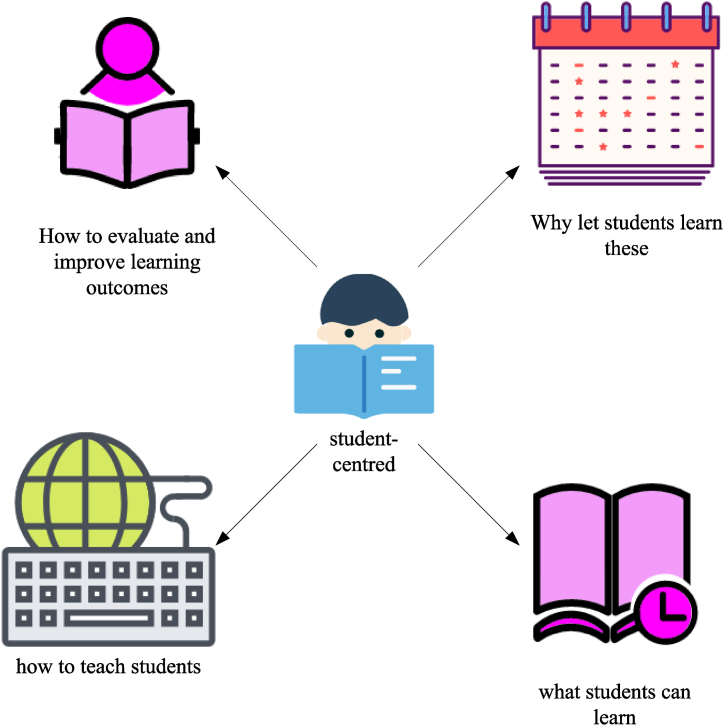
OBE teaching philosophy.

Outcome-based education is an educational philosophy that revolves around four questions: "What can students learn?" "Why do students learn them?" "How do students learn them?" and "How to evaluate and improve learning outcomes", as shown in Fig. 2. It is precisely these four questions that reflect the advantages of outcome-oriented education. These four questions are closely linked to an important theme, "student-centred" [12,13].

What students can learn is the definition and design of learning outcomes, and it is also the beginning of reverse design, which determines the model and method of the entire teaching process. Students learn this because of critical thinking about learning outcomes. The iterative determination of learning outcomes is to make learning outcomes more comprehensive and precise. Learning is to let students understand the impact of the learning outcomes acquired through learning on their future careers and lives and to think about why they should learn from the perspective of students [14,15]. How students learn them refers to the concept of taking learning outcomes as the centre is implemented to implement teaching design, and reasonable and scientific teaching methods and means are adopted to enable students to achieve teaching goals to the greatest extent. Evaluating and improving learning outcomes is to obtain teaching feedback information through several evaluation methods, such as process evaluation and summative evaluation, after implementing teaching activities through reverse-designed teaching design so that the degree of achievement relationship between learning outcomes and expected learning outcomes can be obtained. Using the teaching feedback, a new round of reverse design is carried out to update and improve the implementation details in the teaching so that each student is constantly approaching the final learning outcome and gains a sense of achievement. As the innovation and key point of outcome-oriented education, "reverse design" has played an irreplaceable role in teaching implementation. The talents cultivated by colleges and universities are the main source of social human resources. The reverse design in the OBE concept ensures the scientific and forward-looking training work to a certain extent, which is conducive to improving the quality of talents cultivated in various majors and promoting social progress [16,17].

The construction of a teaching model is a complex and unified process composed of various links, but in the end, they all aim to achieve the same purpose - to achieve learning outcomes. The outcome-based concept is closely related to students and learning outcomes in the whole teaching process and radiates to every link with the two as the centre so that students have more learning gain and a sense of achievement in the learning process [18,19].

In addition, the difference between the outcome-based teaching concept and the traditional teaching concept in the teaching process is reflected in the results and the design method of the entire educational process. The major of PE is to cultivate PE talents with strong knowledge, skills and comprehensive quality for the country, society and schools, so colleges and universities should focus on what kind of talents are needed in the social market and environment and carry out all-round training for students starting from the training results, to make the training process and purpose clearer and clearer. The classroom teaching mode of sports professional air volleyball guided by the OBE concept echoes the training goals of PE professionals, aiming to reversely design the entire teaching specifications of cultivating talents, aiming to reverse the design of the teaching based on the specifications of cultivating talents and aiming at the learning outcomes obtained at graduation, which continuously improves teaching quality with specialised and continuous evaluation methods, to ensure that students achieve their goals at graduation and meet the multiple needs of the new era society for talents [20,21].

### 2.2 GA optimization teaching mode

GA optimization:

Coding the indicators in PE:

Choosing the right fitness: The fitness function is divided into maximum and minimum cases [22,23]. This paper evaluates the system and selects the minimum fitness value, as shown in Equation (1).

(1) $$f_{Normal}\left(x\right)=f_{\max}-f\left(x\right)$$

In Formula 1, $f_{\max}$ represents the maximum value of the fitness function.

Different selection strategies can lead to different selection pressures. The roulette is used [24], and a disc is divided into N parts according to the selection probability $p_{i}$ as given in Equation (2).

(2) $$p_{i}=f_{i}/\sum f_{i}$$

Generating a random number r, see Equation (4), if it satisfies Equation (3), individual i can be selected.

(3) $$\sum_{j=1}^{i-1}p_{j}<r\leq \sum_{j=1}^{i}p_{j}$$

(4) r∈[0,1]

Then, the traditional genetic operator design is adopted.

The optimization function has the following options.

Global maximisation: For the seeking point $x_{\max}\in S$, there are Equations (5), (6)).

(5) $$f\left(x_{\max}\right)\geq f\left(x\right)$$

(6) s.t.∀x∈S

Global minimisation: For the seeking point $x_{\max}\in S$, there are Equations (7), (8)).

(7) $$f\left(x_{\min}\right)\leq f\left(x\right)$$

(8) s.t.∀x∈S

However, the optimization problem can be transformed into a maximum value problem. If the function f finds the minimum value, the transformation is as in Equation (9) and Equation (10),

(9) minf(x)=maxg(x)=max{−f(x)}

(10) g=−f

If the objective function f only takes a negative value and wants to convert it to a positive value, a positive number C can be added, as shown in Equation (11):

(11) maxg(x)→max{g(x)+C}

The specific steps of using GA to extract the characteristics of college students online learning are as follows [25].

Step 1 Keywords are extracted. When learning online, the keywords entered on the system page are recorded as a document set $C_{1}$ as in Equation (12). It is considered to decompose $C_{1}$ into multiple document matrices $A_{1}$ and filter word set B.

(12) $$C_{1}\Rightarrow \left[A_{1}\right]avd\left[B\right]$$

Step 2 Similarly, the test document set $C_{2}$, see Equation (13), $A_{2}$ can be obtained through filtering with B:

(13) $$C_{2}⊳\left[B\right]\left[A_{2}\right]$$

Step 3 $A_{1}$ and $A_{2}$ are combined to get the test matrix A, see Equation (14):

(14) $$\left[A_{1}\right]+\left[A_{2}\right]=\left[A\right]$$

Step 4 In this article, $f\left(a_{i}\right)$ can be used to define function f(a), as in Equation (15), to evaluate the i-th document. Among them, W is the weight of the set B keywords and $W^{\prime }$ is the weight optimised by the GA:

(15) $$f\left(a_{i}\right)=W^{\prime }\times A_{1}$$

Step 5 When Equation (16) is satisfied, or when it is greater than $f\left(C_{2}\right)$ in a certain proportion:

(16) $$f\left(C_{1}\right)>f\left(C_{2}\right)$$

It is assumed that Equation (17) is the document collection in the online learning system of college sports personalization. Equation (18) is the document collection of learners' individual interests or online test evaluation, and Equation (19) is the rest of the test collection. Among them, m≪n.

(17) $$C=\left\{c_{1},\cdots ,c_{n}\right\}$$

(18) $$C_{1}=\left\{c_{1},\cdots ,c_{m}\right\}$$

(19) $$C_{2}=\left\{c_{m+1},\cdots ,c_{n}\right\}$$

B represents the feature set of the learner's information needs, as shown in Equation (20):

(20) $$B=\left\{b_{1},\cdots ,b_{n}\right\}$$

The optimization teaching mode of the genetic algorithm includes the following detailed parameters and optimization process: roulette wheel selection method is used in the selection stage, single-point crossover is used in the crossover stage, and mutation probability is used to mutate individual genes in the mutation stage randomly. The population size is set to a moderate number, and the number of iterations is set to the maximum algebra. The fitness function considers students' academic performance, participation, and the quality of teaching plans. In the optimization process, individual genes constantly evolve through selection, crossover and mutation, aiming at the expected teaching effect. Through continuous iteration, this process gradually makes the teaching mode optimal and improves students' learning experience and comprehensive quality.

## 3 New model of college PE teaching based on flipped classroom and OBE

### 3.1 Construction and implementation of the classroom teaching model of air volleyball

**Fig. 3:**
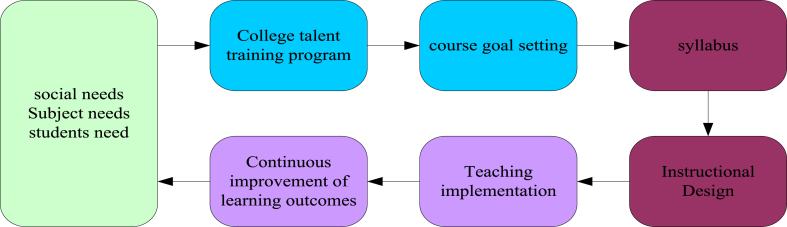
Instructional design under the OBE concept.

The air volleyball classroom teaching model under the OBE concept is a reverse design starting from the final demand. The whole design process was guided by the needs of modern society, disciplines, and students in designing the entire teaching model, as shown in Fig. 3. Various factors in the teaching model were also analysed.

PE teaching mode based on OBE concept (group of OBE):

The classroom teaching model of air volleyball under the OBE concept is a non-instructive teaching mode. In the structure of the teaching process, there are mainly learning stages, such as question raising, trial learning, and cooperative discussion. Teachers use reasonable means and methods to intersperse learning content with exercises. There are three types of teaching organisation.

Teaching model based on the flipped (group of FCM).

(1) Before class: watch teaching videos and communicate with students.
(2) In class: students' learning movement skills independently and teachers' guidance and evaluation.
(3) After class: summarize, reflect, and improve.

Teaching mode based on OBE and flipped classroom (group of OBE + FCM).

(1) Before class: studying in groups, watching videos, and thinking.
(2) In class: team self-studying, stimulating students' active learning, teachers' tour guiding, after-class evaluation.
(3) After class: teachers assign open homework to guide students to think openly and continue to improve.

Traditional teaching mode (group of TTM):

Traditionally, teachers teach, and students practice. Then the class is over.

### 3.2 Experimental design

Testing purposes: The improvement of air volleyball teaching in colleges and universities can be promoted, and the results of new methods of college PE teaching can be discussed by testing and comparing the feasibility and educational impact of the conventional teaching model and the air volleyball classroom teaching mode under the OBE concept.

Expectations before testing: According to the purpose of the research, there are the following two assumptions: (1) The classroom teaching model of air volleyball based on the OBE concept is helpful for students to learn the basic skills and tactics of air volleyball; (2) The classroom teaching model of air volleyball based on the OBE concept can help cultivate students' basic teaching skills and qualities.

The test objects are shown in Table 1.

**Table 1:** Test objects.

group	total	male	female
OBE	10	5	5
FCM	10	5	5
OBE + FCM	10	5	5
TTM	10	5	5

The test subjects were 40 students who had chosen to major in air volleyball at Hebei Normal University for Nationalities. First of all, psychologically, college students' physical and mental development tended to mature. They could think and judge independently and control their attention more stably, with strong self-restraint abilities. The students also had basic sports ability. According to the project characteristics of air volleyball, students were particularly interested in the holistic practice of air volleyball learning [26]. College students' psychological maturity and various abilities, including independent thinking, judgment, attention control, self-discipline, and basic sports ability. This helps to establish a learning environment so that students can better participate in physical education teaching activities, improve the teaching effect, and promote the positive learning experience in balloon volleyball class by combining psychological maturity and sports ability. Therefore, in teaching, it was necessary to let students combine physical exercise and logical thinking well, provide students with an opportunity to learn professional knowledge and innovative learning methods and improve the teaching effect. In teaching, according to the age characteristics of students, various teaching methods should be adopted, and multiple methods such as discussion method, intuitive method and cooperative learning should be used to mobilise students' enthusiasm for learning and practice [27].

The sample selection process has been carefully designed. First, the researcher selected students majoring in balloon volleyball at a university as potential research subject. This choice is based on the purpose of the study, that is, to evaluate the influence of the new teaching mode on balloon volleyball education.

From the potential research objects, the stratified sampling method is adopted to ensure the diversity and representativeness of the samples. The levels of stratified sampling include different teaching modes, such as OBE, FCM, OBE + FCM and TTM. In each teaching mode, 50 % male and 50 % female students are selected to keep the gender balance.

Finally, 40 students' samples were obtained through this sample selection process, covering different teaching modes and genders. This sample selection method is helpful in ensuring the reliability and validity of the research results to comprehensively evaluate the influence of varying teaching modes on students' learning achievements.

### 3.3 Test results

Reference 28 established a teaching model based on the theory of multiple intelligences and took university sports basketball teaching as the research object, incorporating students' sports skills, practical abilities, and cognitive abilities for teaching effectiveness analysis [28]. Reference 29 proposed a pattern-based practical physical education teaching method and conducted a survey on the learning effectiveness of students of different age groups using the proposed method through semi-structured interviews [29]. Among them, sports skills, practical abilities, and cognitive abilities can more comprehensively reflect students' comprehensive quality and development. Semi-structured interviews can collect more detailed and specific feedback from students, helping evaluators thoroughly understand teaching effectiveness. Therefore, this article has cited and improved the evaluation indicators and methods of references 28 and 29. Before the implementation of teaching, this article divides the testing indicators into cognitive, physical, and technical categories. Cognitive categories are surveyed through interviews. In the category of physical fitness, the 5-m triathlon and approach touch are used as test indicators. The physical and technical categories selected self-buffering as the testing content to investigate and test the experimental subjects. In the testing and evaluation, this article sets the scoring range to 1–10 points and statistically analyses the performance differences between the two groups of students through a T-test.

**Fig. 4:**
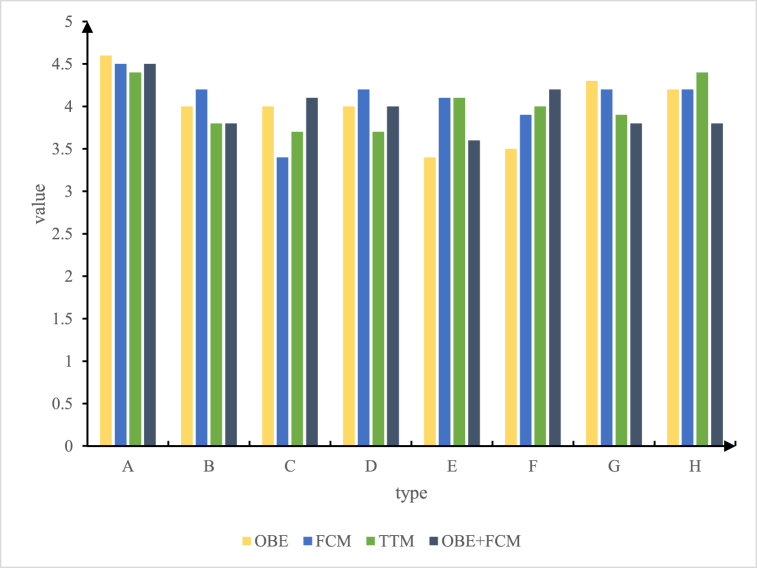
The cognitive situation of students in each group.

Results and analysis of students' cognitive situation:

The differences in students' cognitive situation are shown in Table 2, and the detailed cognitive situation of each group is shown in Fig. 4.

**Table 2:** Students' cognitive situation.

numbering	analysis items	p
A	Did you know the sport of air volleyball before?	0.912
B	Have you ever learned air volleyball before?	0.854
C	How much do you like air volleyball?	0.654
D	Do you discuss air volleyball with your classmates and friends?	0.652
E	How well do you understand the theoretical knowledge of air volleyball?	0.346
F	Your mastery of air volleyball skills and tactics	0.567
G	Do you like watching air volleyball games?	0.346
H	Have you ever participated in a school air volleyball activity or competition?	0.631

The study examined the cognitive abilities of students using a questionnaire format to ensure that there were no differences in test subjects before starting teaching. The results of the air volleyball cognition survey of the students in the experimental group and the control group are shown in Table 2. It can be said that there was no significant difference between the groups in terms of student's understanding of air volleyball. That is, the test subjects used to analyse the relevant indicators and phenomena might be students of male and female courses in the control group and the experimental group.

Test results and analysis of students' physical fitness.

**Fig. 5:**
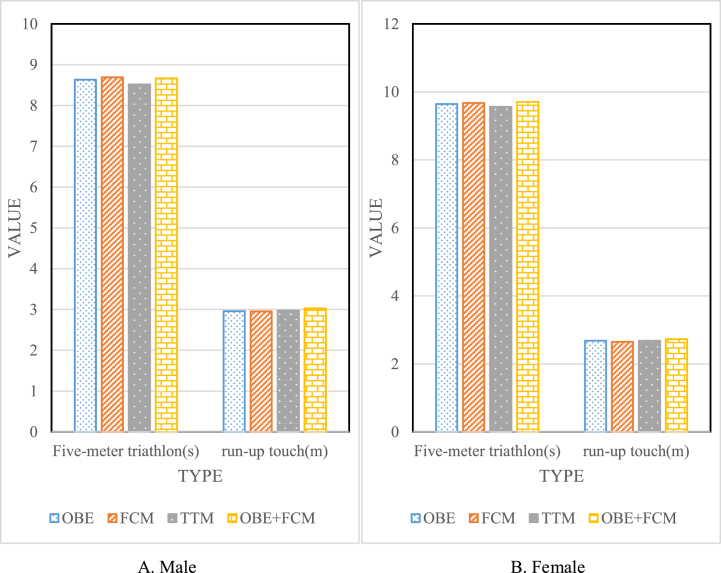
Pre-test of physical fitness in each group. (A) for males and (B) for females.

Before the implementation of this teaching, the contents of the physical fitness test for the students were run-up touch height, three items of 5 m. After the test, the test results were compared and analysed by the indifference test (T-test). The results are shown in Fig. 5. As can be seen from Fig. 5A, the average of the test results of three items of 5 m in each group of males was about 8.6s, and the score of run-up touch height was about 3 m, with no significant difference. As can be seen from Fig. 5B, the average of the test results of three items of 5 m for each group of females was about 9.7s, and the score of run-up touch height was about 2.6 m, with no significant difference.

Test results and analysis of basic skills.

**Fig. 6:**
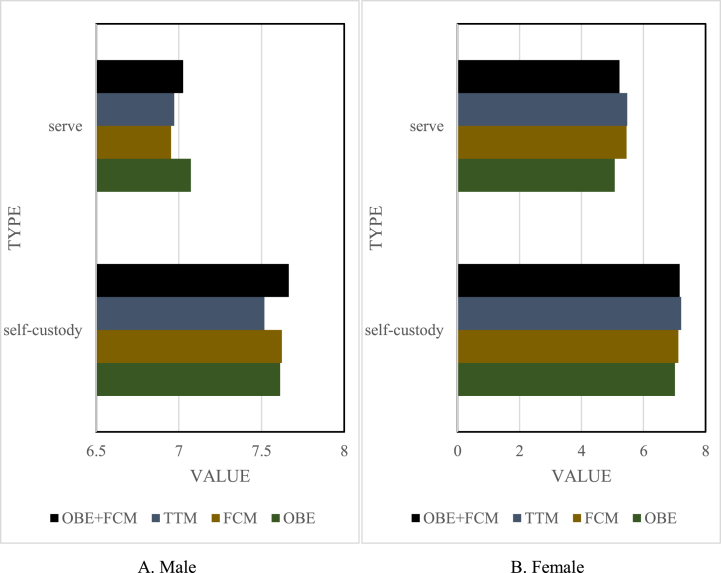
Basic technical pre-test of each group. (A) for males and (B) for females.

Before the implementation of teaching, the basic skills of air volleyball were selected to test the two contents of serving and self-cushion, and the T-test was carried out on the average value of the test results of each group. The results are shown in Fig. 6, and the specific results are shown in Fig. 6A and B. There was little difference between the number of self-cushions and servings between the male' and female' groups. It can be concluded that the air volleyball cushioning and serving skills of each group in the male and female classes were at the same level, with no significant difference, and the test conditions were met.

Test results and analysis of students' cushioning skills.

**Fig. 7:**
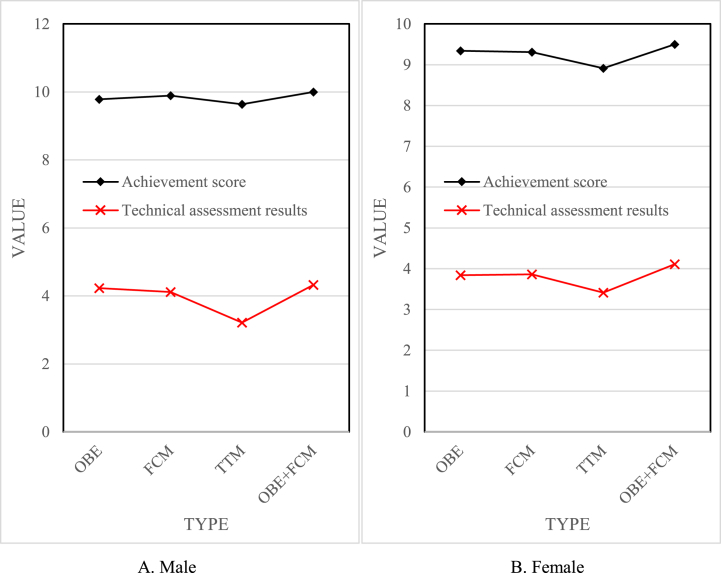
Post-test of each group of ball cushioning skill. (A) for males and (B) for females.

As seen from Fig. 7, compared with the traditional model, the scores of the other groups all increased. As shown in Fig. 7A, the males' OBE + FCM group achieved the standard score of 10; the OBE group was 9.78, the FCM group was 9.89, and the TTM group was 9.638. The OBE + FCM group's up-to-standard score was 3.8 % higher than that of the TTM group. The technical assessment score of the males' OBE + FCM group was 4.325, the OBE group was 4.231, the FCM group was 4.112, and the TTM group was 3.216. The OBE + FCM group's technical assessment score improved by 34.5 % compared with the TTM group. As shown in Fig. 7B, the females' OBE + FCM group achieved the standard score of 9.5; the OBE group was 9.342; the FCM group was 9.311, and the TTM group was 8.911. The OBE + FCM group achieved a 6.6 % improvement in the achievement of the standard compared with the TTM group. The technical assessment score of the females' OBE + FCM group was 4.112, the OBE group was 3.842, the FCM group was 3.863, and the TTM group was 3.411. The technical evaluation score of the OBE + FCM group was 20.6 % higher than that of the TTM group. It can be found that whether it was a male or a female, the OBE + FCM group had the highest score, and the TTM group had the lowest score. This showed that the students in the OBE + FCM group and the TTM group had significant differences in the application and mastery of the skill in the ball-cushion skill after the experiment. In the teaching link of the ball-cushion skill during the experiment, in the OBE + FCM group, the skill of air volleyball was used in the form of technical application to teach the technical movements of the ball, create a situation, and imitate the receiving and serve in the game to carry out the teaching and organisation of the ball. In contrast, the TTM group taught in the form of traditional education, explaining-demonstration-exercise-correction. According to the requirements of the teaching syllabus, two-person padding skills are used in the assessment of ball padding skills, which mainly tests the student's mastery of the hand shape and coordination of paddling skills, as well as their coordination with preparation postures and movements.

Test results and analysis of student passing skills.

**Fig. 8:**
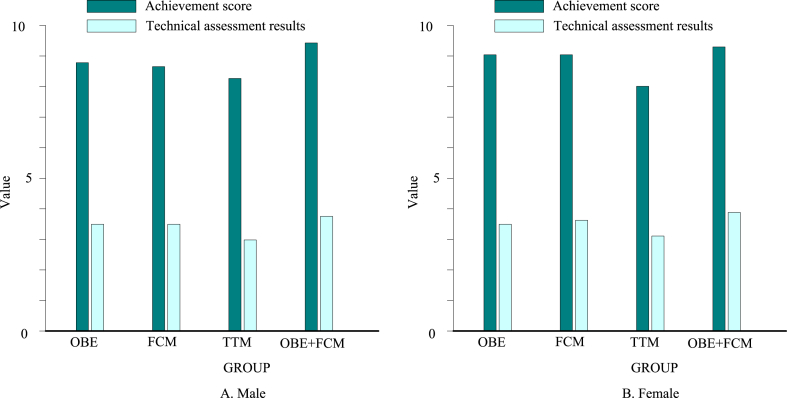
Post-test of passing skills in each group. (A) for males and (B) for females.

As shown in Fig. 8, the passing skills of each group still showed the trend of the lowest in the TTM group and the highest in the OBE + FCM group. As shown in Fig. 8A, the achievement of the males' OBE + FCM group was 14.3 % higher than that of the TTM group, and the males' OBE + FCM group's technical assessment performance was 42.8 % higher than that of the TTM group. As shown in Fig. 8B, the achievement of the females' OBE + FCM group was 14.5 % higher than that of the TTM group, and the technical assessment performance of the females' OBE + FCM group was 29.3 % higher than that of the TTM group. After 14 weeks of teaching, students in each group had a basic grasp of the basic skills of air volleyball, but there was still room for improvement in their understanding and application of skills. From Fig. 8, it can be concluded that there were significant differences in the passing skills between the new model of PE teaching and the traditional model in the male and female classes after teaching activities. The experimental group was higher than the control group regarding passing skills and technical evaluation. Passing in air volleyball is a basic skill and the most critical technical action, which plays a vital role in actual combat, which is a difficult skill for beginners to master. In the classroom conversation, some students reported that after the whole semester of study, they still felt that they had only learned the basic movement essentials for passing skills and could complete the movements but could not use them flexibly in actual combat. Therefore, in this semester's teaching, the students' passing skills and technical assessment results belonged to the normal range of beginners.

Results and analysis of students' spiking skills test.

**Fig. 9:**
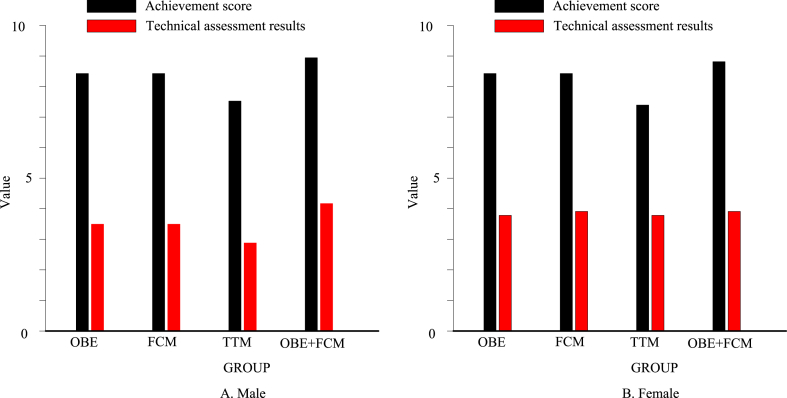
Post-test of spiking skill in each group. (A) for males and (B) for females.

As shown in Fig. 9, the passing skills of each group still showed the trend of the lowest in the TTM group and the highest in the OBE + FCM group. As shown in Fig. 9A, the achievement of the males' OBE + FCM group was 20.8 % higher than that of the TTM group, and the technical assessment performance of the males' OBE + FCM group was 38.9 % higher than that of the TTM group. As shown in Fig. 9B, the achievement of the females' OBE + FCM group was 0.2 % higher than that of the TTM group, and the females' OBE + FCM group's technical assessment performance was 2 % higher than that of the TTM group. It can be concluded that after 14 weeks of practice of the OBE concept and the flipped classroom air volleyball classroom teaching model, the technical and technical assessment performance of the experimental group in the male class is better than that of the control group in the traditional classroom teaching model, while there was no significant difference in the average achievement and technical assessment scores between the experimental group and the control group in the female class. This paper believed this was closely related to females' sports experience and physical quality, and males had certain advantages over females in terms of sports experience and physical quality. In addition, the spiking technical action in air volleyball is difficult to master in this sport, and it has high requirements in terms of both ball and coordination. However, from the test data, the male experimental group had obvious differences in spiking skills compared to the control group, not only in the success rate but also in the technical evaluation, which was inseparable from their daily exercise habits and specialities. Some students specialised in basketball, with good bounce and explosiveness, while others specialised in badminton, with good speed and coordination. These factors have a certain degree of influence on the learning and mastery of air volleyball skills.

Results and analysis of students' serving skills test.

**Fig. 10:**
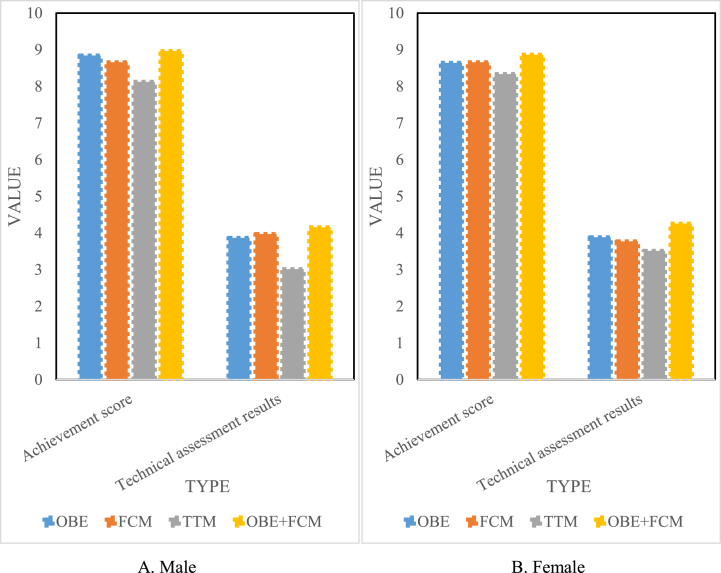
Post-test of each group's serving skill. (A) for males and (B) for females.

As shown in Fig. 10, the passing skills of each group still showed the trend of the lowest in the TTM group and the highest in the OBE + FCM group. As shown in Fig. 10A, the achievement of the males' OBE + FCM group was 10.3 % higher than that of the TTM group, and the males' OBE + FCM group's technical assessment performance was 37.9 % higher than that of the TTM group. As shown in Fig. 10B, the achievement of the females' OBE + FCM group was 6.3 % higher than that of the TTM group, and the females' OBE + FCM group's technical assessment performance was 21.1 % higher than that of the TTM group. There was a significant difference between the OBE + FCM and TTM groups in the service test scores, especially in the technical evaluation scores. The experimental group using the outcome-based classroom teaching model was better than the control group in the traditional classroom teaching mode regarding the accuracy and completion quality of serving technical movements. Serving is a basic skill in air volleyball and plays a vital role in the game. In the teaching process, teaching based on the outcome-based concept enables students to better understand the details and internal laws of technical movements in the learning process, helping students establish more accurate movement representations and concentrate more. Moreover, learners can conduct trial teaching as teachers throughout the whole teaching process. Through learning and applying movement skills in different identities, learners can have a richer experience, establish a sense of self-identity, and have a deeper understanding of the teaching content.

It can be seen that OBE flipping topics can effectively improve students' physical education skills. This is because OBE encourages personalised learning and adjusts teaching methods according to student's needs and abilities. In physical education teaching, educators can develop customised training plans based on students' exercise levels and interests so that each student can progress at a level that suits them. OBE emphasises the evaluation of students' actual performance and provides timely feedback. Physical education teaching, this can be evaluated by observing students' motor skills, physical fitness level, and teamwork. Timely feedback can help students understand their strengths and the direction of improvement.

Results and analysis of students' lesson plans:

Compiling lesson plans can highlight students' understanding of air volleyball and combine PE compulsory theoretical knowledge and skills with sports learning, which is conducive to students' acceptance and learning of skills. At the same time learning a sports skill mastering the teaching methods and means of this sports skill is the best way to cultivate college PE skills. After learning, students can not only gain a motor skill but also master its teaching method, which is helpful for students to understand and identify themselves. Students can give full play to their strengths, complete the writing of lesson plans independently, and improve their problem-solving ability.

Table 3 shows the writing scores of the lesson plans. The OBE + FCM group has the largest number of students with a score of 85 and above and the lowest number of students with a score of 60–71. This showed that the air volleyball classroom teaching mode based on the OBE concept and flipped classroom can enable students to combine practice and theory and let most students master the skills of writing lesson plans, giving most students a deeper understanding of air volleyball.

**Table 3:** Number of students scoring each section of the lesson plan.

fractional segment	60–71	72–84	>84
OBE	4	4	2
FCM	3	5	2
TTM	7	2	1
OBE + FCM	3	4	3

Results and analysis of students' test lecture scores:

The ability of students to test lectures reflects the student-centred teaching philosophy.

As shown in Table 4, the number of students in the OBE + FCM group with a high score of 85 or above was two, and the number of students with a score of 60–71 was the lowest. This showed that the design and distribution of teaching content in the teaching process is the main factor affecting students' learning outcomes. In the traditional classroom teaching model, the teaching content of teachers only includes the mastery of sports skills and competition requirements, etc., and the teaching skills of this project are not gradually taught to students. There is no special training for students' teaching skills either. Suppose teachers do not train students' teaching skills in the teaching content setting or do not get sufficient exercise. In that case, students may have serious problems in employment during the transition period of "learning to teach". Mock lectures exercise students' ability to apply learning outcomes, which requires students to learn through the theoretical knowledge of various school courses, put the theory into practice, and constantly polish and temper. In the evaluation link of the mock lecture, it can be known that the air volleyball classroom teaching mode based on the OBE concept and flipped classroom meets the needs of students' ability to survive and work in the future and helps students improve their personal, professional quality and ability.

**Table 4:** Number of students who scored each paragraph in the trial lecture.

fractional segment	60–71	72–84	>84
OBE	4	4	2
FCM	3	5	2
TTM	6	3	1
OBE + FCM	2	6	2

Students have a positive experience of the OBE concept and flip classroom mode, and they think that the classroom is more interesting by observing videos to understand skills in advance. In contrast, in the traditional mode, students are bored and lack interest in practice. This feedback reveals the positive experience triggered by the new teaching model, which provides a useful reference for educational reform.

To better adapt to specific sports or sports activities. First, we must consider the characteristics of sports and learning needs to determine the teaching objectives. Then, the teaching content and methods must be adjusted to ensure they meet the technical requirements of different sports. For example, ball games may focus on teamwork, while individual events may focus more on particular skills. In addition, the teaching mode should consider the difficulty of sports and the age group of students. By flexibly adjusting teaching strategies and contents, this model can adapt to various sports or sports activities and improve students' learning effects in different sports fields.

## 4 Discussion

This paper compares the conventional teaching mode with the classroom teaching mode of balloon volleyball based on the OBE concept and deeply studies the teaching effect. In the experimental design, students' cognition, physical quality, and technology are comprehensively tested, which provides detailed data that supports a comprehensive evaluation of the teaching effect. Secondly, through the innovation of teaching mode, the concept of OBE and the elements of flipping the classroom are introduced, and the theoretical knowledge and practical skills are combined to enhance the depth and breadth of students' learning. In addition, the student's ability to understand and apply the new teaching model is demonstrated through the grading results of the students' teaching plans and trial lectures, which provides a feasible way to cultivate students' teaching skills. Most importantly, the personalised learning model based on a genetic algorithm has injected new ideas into the field of physical education and explored new ways to improve the teaching effect and cultivate students' comprehensive quality by optimising teaching modes to meet students' learning needs individually. Therefore, this study not only expands the research field of physical education teaching in theory but also substantially contributes to improving teaching quality and cultivating students' all-round quality in practice.

## 5 Conclusions

By comparing the conventional teaching mode with the classroom teaching mode of balloon volleyball based on the OBE concept, this paper deeply analyses the influence of different teaching methods on students' cognition, physical fitness and technology. It provides empirical data for physical education teaching. Innovatively introducing the OBE concept and flipping classroom elements, the teaching mode is optimised, theoretical knowledge and practical skills are better integrated, and the depth and breadth of students' learning are improved. Through the results of students' teaching plans and trial lectures, the students' understanding and ability to apply the new teaching model is demonstrated, which provides a new way to cultivate students' teaching skills. The applicability of the article to different sports needs further verification, and the research duration is only one semester, lacking long-term effect verification. Future research can consider a longer follow-up to deeply understand the effect of the new teaching mode on students' long-term development. Such research will help fully understand educational reform's long-term impact on students' comprehensive literacy.

## Ethics statement

This study was approved by the Ethics Committee of Hebei Normal University for Nationalities, the participants were consented by an informed consent process that was reviewed by the Ethics Committee of Hebei Normal University for Nationalities and certify that the study was performed in accordance with the ethical standards as laid down in the 1964 Declaration of Helsinki.

## Data availability statement

The experimental data used to support the findings of this study are available from the corresponding author upon request.

## Funding statement

We confirm that no funding has been received from any funds.

## Consent to participate

Not applicable.

## CRediT authorship contribution statement

**Yanjun Kong:** Writing – review & editing, Writing – original draft, Resources, Project administration, Investigation, Conceptualization. **Weihong Wang:** Formal analysis, Data curation. **Bakhrom Rajabov:** Visualization, Validation, Software.

## Declaration of competing interest

The authors declare that they have no known competing financial interests or personal relationships that could have appeared to influence the work reported in this paper.
